# False-negative rate in the extended prospective TATTOO trial evaluating targeted axillary dissection by carbon tattooing in clinically node-positive breast cancer patients receiving neoadjuvant systemic therapy

**DOI:** 10.1007/s10549-022-06588-2

**Published:** 2022-04-22

**Authors:** Jana de Boniface, Jan Frisell, Thorsten Kühn, Ingrid Wiklander-Bråkenhielm, Karin Dembrower, Per Nyman, Athanasios Zouzos, Bernd Gerber, Toralf Reimer, Steffi Hartmann

**Affiliations:** 1 Department of Molecular Medicine and Surgery, Karolinska Institutet, Stockholm, Sweden; 2 Department of Surgery, Breast Centre, Capio St. Göran’s Hospital, Mariebergsporten 2, 11219 Stockholm, Sweden; 3 Department of Breast, Endocrine and Sarcoma Surgery, Karolinska University Hospital, Stockholm, Sweden; 4 Department of Obstetrics and Gynecology, Klinikum Esslingen, Esslingen, Germany; 5 Department of Radiology, Capio St. Göran’s Hospital, Stockholm, Sweden; 6 Department of Physiology and Pharmacology, Karolinska Institutet, Stockholm, Sweden; 7 Department of Surgery, Skaraborg Hospital, Lidköping, Sweden; 8 Department of Mammography, Karolinska University Hospital, Stockholm, Sweden; 9 Department of Oncology and Pathology, Karolinska Institutet, Stockholm, Sweden; 10 Department of Obstetrics and Gynecology, University of Rostock, Rostock, Germany

**Keywords:** Breast cancer, Lymph node metastasis, Targeted axillary dissection, Neoadjuvant chemotherapy

## Abstract

**Purpose:**

In clinically node-positive breast cancer patients receiving neoadjuvant systemic therapy (NST), nodal metastases can be initially marked and then removed during surgical axillary staging. Marking methods vary significantly in terms of feasibility and cost. The purpose of the extended TATTOO trial was to report on the false-negative rate (FNR) of the low-cost method carbon tattooing.

**Methods:**

The international prospective single-arm TATTOO trial included clinically node-positive breast cancer patients planned for NST from November 2017 to January 2021. For the present analysis, patients who received both the targeted procedure with or without an additional sentinel lymph node (SLN) biopsy *and* a completion axillary lymph node dissection (ALND) were selected. Primary endpoint was the FNR.

**Results:**

Out of 172 included patients, 149 had undergone a completion ALND. The detection rate for the tattooed node was 94.6% (141 out of 149). SLN biopsy was attempted in 132 out of 149 patients with a detection rate of 91.7% (121 out of 132). SLN and tattooed node were identical in 58 out of 121 individuals (47.9%). The combined procedure, i.e. targeted axillary dissection (TAD) was successful in 147 of 149 cases (98.7%). Four out of 65 patients with a clinically node-negative status after NST had a negative TAD but metastases on ALND, corresponding to a FNR of 6.2%. All false-negative TAD procedures were performed in the first 2 years of the trial (2018–2019, *p* = 0.022).

**Conclusion:**

Carbon tattooing is a feasible marking method for TAD with a high detection rate and an acceptably low FNR.

The TATTOO trial was preregistered as prospective trial before initiation at the University of Rostock, Germany (DRKS00013169).

## Introduction

In initially clinically node-positive (cN +) breast cancer, conversion to pathological node negativity (ypN0) through neoadjuvant systemic therapy (NST) occurs in 13–60% depending on breast cancer subtype [1]. Since the proportion of ypN0 patients, i.e. those achieving an axillary pathologically complete response (pCR) will most probably keep rising, it is highly relevant to individualize and de-escalate axillary staging strategies. Whilst cN + patients commonly underwent an axillary lymph node dissection (ALND) after NST regardless of individual axillary response, most modern guidelines recommend sentinel lymph node biopsy (SLNB), targeted lymph node biopsy (TLNB) and/or a combination of both (targeted axillary dissection, TAD) [2, 3] in an attempt to reduce unnecessary arm morbidity. The concept of targeted techniques evolved from the need to improve detection rates and false-negative rates (FNRs) which are unacceptably high (17%) when SLNB alone is used after NST in cN + patients [4]. With TLNB or TAD, however, pooled FNRs drop to 6.28% and 5.18%, respectively [5]. The oncological consequence of false negativity in this situation is still unclear: the omission of ALND after a negative SLNB alone does not appear to translate into increased recurrence rates [6], and recurrence or survival data in case of a negative TLNB or TAD as the single axillary staging surgery are still lacking. The ongoing prospective EUBREAST-03/AXSANA study will hopefully be able to clarify the oncological significance of different post-NST axillary staging procedures in initially cN + patients converting to ycN0 [7].

There is a multitude of marking techniques with various advantages and disadvantages concerning patient comfort, detection rates, necessity of a preoperative second localization procedure, technical requirements and cost. The use of iodine seeds such as described in the RISAS trial [8] is hindered by legislation regarding radioactive compounds in several countries, magnetic seeds hamper the use of magnetic resonance imaging during PST [9], and metal clips, necessitating a second pre- or intraoperative localization procedure, had a low detection rate in prospective feasibility trials [10, 11]. Tattooing lymph node metastases with purified carbon dye is relatively simple and low-cost (36 Euro per procedure), and has been reported to render high detection rates [12]. Most reports strictly focus on feasibility in very small populations [13–17]; the calculation of a FNR however depends on the performance of a completion ALND, and has only been evaluated in two reports. Firstly, the TATTOO trial’s primary analysis analysed 60 ycN0 patients in whom TAD was successful and reported a FNR of 9.1% [18]. Secondly, Park et al. reported on 20 patients, 10 of whom had a so-called modified SLNB, implying that a tattooed node and SLN were removed at the same time, followed by an ALND. Here, the FNR was 0% [13]. Today, ALND is omitted in case of a negative axillary staging procedure, and data on FNR are thus difficult to obtain but highly important to take into consideration.

Thus, the aim of the present analysis was to report on the FNR of the tattooing technique in the prospective TATTOO trial which has been substantially expanded to form the largest prospective cohort of TAD procedures yet reported testing carbon tattooing in combination with completion ALND.

## Methods

The multicentre prospective TATTOO trial enrolled clinically node-positive breast cancer patients at two German and three Swedish sites between November 2017 and October 2019. After that date, only Swedish sites continued enrolment until January 2021 in order to obtain a larger sample size regarding one of the secondary outcomes, FNR. Inclusion criteria were written informed consent, patient age of 18 years or more, histological confirmation of a unilateral or bilateral invasive breast cancer and planned NST. Histopathological or cytological confirmation of clinically and/or sonographically suspected ipsilateral axillary lymph node metastases was not mandatory but always obtained at Swedish sites. Patients with inflammatory breast cancer or confirmed distant metastases were not eligible. The primary endpoint of the TATTOO trial, intraoperative detection rate, as well as secondary endpoints such as concordance rate between SLNB and TLNB, complication rates and FNR in the first 110 patients eligible for analysis, have been published previously [18].

Prior to NST, suspicious axillary lymph nodes were biopsied by core needle or fine needle aspiration. The largest and/or biopsy-confirmed metastastic node was then injected with highly purified carbon suspension either at the time of biopsy or at a separate session. Carbon suspension was deposited into the nodal cortex and the surrounding tissue, and the total injected volume was recorded. If possible, physicians could also register the separate volumes injected into the lymph node cortex, the soft tissue surrounding the lymph node and the track leading from lymph node to skin. The creation of a track of carbon suspension from the node towards the nearest skin surface was optional, as was the intentional tattooing of the overlying dermis. Both Spot® (GI Supply, Camp Hill, Pennsylvania, USA) and CARBO-REP® (Sterylab, Rho/Milan, Italy) were used.

Following NST, all included patients underwent TLNB, i.e. the removal of the marked lymph node/s by intraoperative visual identification. Completion ALND included axillary levels I and II but was not mandatory in this trial but was indicated in accordance to national guidelines: in Germany, a completion ALND was optional in patients with a negative staging procedure whilst in Sweden, completion ALND was still mandatory in all patients at the time of the trial. SLNB was undertaken at the discretion of the responsible surgeon and comprised the preoperative injection of radio-labelled technetium and/or blue dye. No injection of isosulfan was undertaken. No specific training was required from surgeons active in the trial.

For the present analysis, only included patients who had received a completion ALND were selected in order to be able to calculate the FNR. In order to verify the presence of lymphatic tissue in the TLNB specimen, data collected in the case report form (CRF) were completed with data from pathology reports obtained for all patients. Likewise, surgical reports were scrutinized to obtain information whether the TLN was retrieved before, during or after ALND.

Histopathological assessment of resected nodes was performed according to institutional routine using standard haematoxylin and eosin staining. Immunohistochemical staining aiming to detect isolated tumour cells was not mandatory according to protocol. Axillary pathological complete response (pCR) was confirmed if no tumour cells were present in any resected axillary lymph node, i.e. isolated tumour cells were defined as non-pCR. Breast pCR was defined as the absence of residual invasive tumour in the breast, i.e. residual in situ disease only was classified as breast pCR. Both surgeons and pathologists were asked to rate the surgical staging procedure and the histopathological assessment, respectively, as “easy” or “difficult”. Physicians performing pre- and post-NST ultrasound of the axilla (gynaecologists in Germany, radiologists in Sweden) were requested to document the number of suspicious lymph nodes, the size of the tumour and the largest lymph node metastasis, and radiological features of the marked nodal metastasis (Solbiati Index, cortex thickness, hilus appearance and suspicious infra- or supraclavicular nodes) before and after NST.

The trial obtained ethical permission in Germany and Sweden (2018/841-31/2) and was performed in line with the principles of the Declaration of Helsinki. All participants gave their informed written consent prior to trial entry. The trial was preregistered as prospective trial before initiation at the University of Rostock, Germany (DRKS00013169).

### Statistical analysis

Descriptive frequencies are presented as numbers and percentages or as median values (minimum–maximum). Detection rate was defined as the number of cases with a successful identification of at least one tattooed node during surgery divided by all analysed cases. FNR was defined as the number of negative TAD procedures divided by all cases with metastastic deposits (at least isolated tumour cells) found on ALND. For the assessment of the agreement between clinical and histopathological nodal stage after NST, Cohen’s kappa coefficient (*κ*) was calculated, which is interpreted as poor (*κ* < 0.00), slight (0.00 ≤ *κ* ≥ 0.20), fair (0.21 ≤ *κ* ≤ 0.40), moderate (0.41 ≤ *κ* ≤ 0.60), substantial (0.61 ≤ *κ* ≤ 0.80) or almost perfect (*κ* > 0.80) agreement. For the evaluation of the distribution of categorical variables in a 4 × 4 cross table, a chi-squared (*χ*^2^) test was performed. For all analysis, SPSS® version 28 (IBM, Armonk, New York, USA) was used. The significance level was set at *p* < 0.05 for all tests.

## Results

**Fig. 1:**
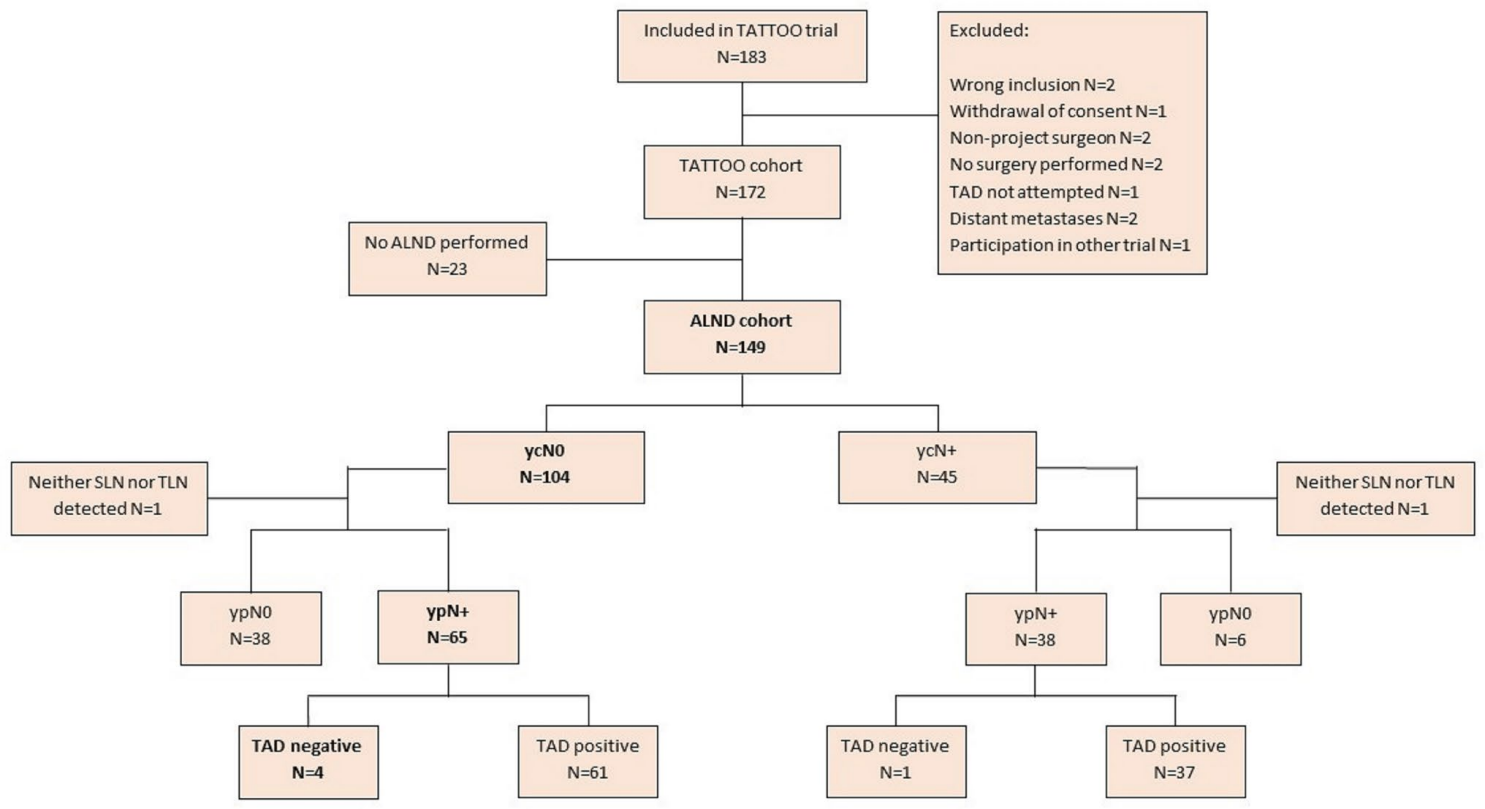
Flow chart for inclusion into the TATTOO trial and selection of population for the present analysis

Between November 2017 and June 2020, 183 patients were prospectively enrolled in the TATTOO trial; 11 were excluded (see Fig. 1). A completion axillary lymph node dissection (cALND) was performed after TLN biopsy/TAD in 149 eligible patients (86.6%) who thus entered the present analysis. For patient and tumour characteristics, see Table 1.

**Table 1:** Patient and tumour characteristics of all patients included in the TATTOO trial who underwent completion axillary lymph node dissection (*N* = 149)

Site	*N* (%)
Capio St. Göran’s Hospital, Sweden	78 (52.3)
University Hospital Rostock, Germany	35 (23.5)
Esslingen Breast Clinic, Germany	14 (9.4)
Karolinska University Hospital, Sweden	19 (12.8)
Skaraborg Hospital, Sweden	3 (2.0)
Age (years)^a^	56 (28–79)
Body Mass Index^a^	25.5 (18–41)
Breast surgery	
Mastectomy	54 (36.2)
Breast-conserving surgery	95 (63.8)
Clinical tumour stage at diagnosis	
cT1	23 (15.4)
cT2	98 (65.8)
cT3	24 (16.1)
cT4	4 (2.7)
Clinical nodal stage at diagnosis	
cN1	121 (81.2)
cN2	27 (18.1)
cN3	1 (0.7)
Tumour multicentricity	
Yes	34 (22.8)
No	115 (77.2)
Histological tumour type	
Ductal	133 (89.3)
Lobular	10 (6.7)
Mixed ductal and lobular	2 (1.3)
Others	4 (2.7)
Tumour subtype	
HR + HER2 −	63 (42.3)
HR + HER2 +	30 (20.1)
HR-HER2 +	19 (12.8)
HR-HER2-	37 (24.8)
Nottingham histological grade	
1	0 (0.0)
2	58 (38.9)
3	90 (60.4)
Missing	1 (0.7)
Proliferation (Ki67, %)^a^	40.0 (6–100)
Number of suspicious axillary lymph nodes on pre-treatment ultrasound^a^	2 (1–15)
Maximum size of largest axillary metastasis on pre-treatment ultrasound (mm)^a^	19.5 (7–56)
Tumour size on pre-treatment ultrasound (mm)^a^	30 (11–134)

*HR* hormone receptor

^a^Median (minimum–maximum)

For carbon tattooing, a median volume of 0.5 ml (0.10–5 ml) of CARBO-REP® (in 114 of 149, 76.5%) or Spot® (in 35 of 149, 23.5%) was injected into the largest metastastic lymph node and surrounding tissue. Because of difficulties separating specific volumes injected into nodal cortex and surrounding tissue, data on these separate injection locations were missing in 70.5 and 71.8%, respectively, and are therefore not reported here. A track of carbon suspension between lymph node and dermis was created in 113 patients (75.8%), and a skin tattoo added in 96 patients (67.6%). The median distance from lymph node to skin was 20 mm (5–42 mm). In four cases an adverse event was registered, consisting of unintentional skin tattooing. The tattooed TLN was identified in all but eight cases (detection rate 94.6%); in five cases with successful detection, however, the theatre report revealed that TLNs were identified during but not before ALND. In 12 cases, the excised tattooed tissue did not contain any lymphoid structures and in one additional case only capsular structures. Sentinel lymph node (SLN) biopsy was attempted in 132 out of 149 patients with a SLN detection rate of 91.7% (121 out of 132). The SLN was identical with the targeted lymph node (TLN) in 58 out of 121 individuals (47.9%). The combined TAD procedure was successful in detecting a SLN and/or TLN in 147 of 149 cases (98.7%).

Post-treatment clinical nodal status was ycN0 in 104 out of 149 patients (69.8%), 38 of whom (36.5%) were confirmed to be node negative by histopathological evaluation (kappa-value 0.168, slight agreement). TAD contained only ITC in 11 ycN0 cases, seven out of whom had no further metastases on ALND (63.6%). In 26 out of 65 ypN + cases (40.0%) with successful TAD, ALND revealed no further metastases than those detected on TAD. Four out of these 65 ycN0 patients with a positive ypN status had a negative TAD, corresponding to a FNR of 6.2% (Fig. 1). In two of these four cases, the TLN was found to not contain any lymphoid tissue on histopathological evaluation and may thus have been a failed TLN procedure; in the other two cases, ALND revealed only one lymph node containing isolated tumour cells and micrometastasis, respectively. No significant association between clinical data and false negativity was found. For details regarding ycN0 false-negative cases, see Table 2. Since all false-negative TAD procedures were performed in the first 2 years of the trial (2018–2019, *p* = 0.022), a learning curve may be suspected. When additionally considering 44 ycN + patients with a successful TAD procedure for FNR, one case was found to be false negative. Six out of 44 ycN + patients had no residual nodal metastases on histopathology. The overall FNR including both ycN + and ycN0 cases was 4.9%.

**Table 2:** Characteristics of four false-negative cases amongst the population classified as clinically node negative after neoadjuvant systemic therapy (NST)

	Age (years)	BMI (kg/m^2^)	Clinical tumour stage	Clinical nodal stage	Number of suspicious nodes pre-NST	Injected volume CARBO-REP® (ml)	SLN = TLN	Number of metastases on ALND	TLN lymphoid tissue
1	56	36.8	1	1	3	3.0	No	1 (mic)	Yes
2	52	23.3	1	1	1	0.6	Yes	1 (ITC)	Yes
3	44	25.0	3	1	1	0.6	No	1 (mac)	No
4	76	33.3	4	2	4	1.0	No	1 (mac)	No

*SLN* sentinel lymph node, *TLN* targeted lymph node, *NST* neoadjuvant systemic therapy, *ITC* isolated tumour cells, *Mic* micrometastasis, *Mac* macrometastasis, *BMI* Body Mass Index, *ALND* axillary lymph node dissection

## Discussion

In this large prospective cohort of 149 breast cancer patients from the TATTOO trial, undergoing both TAD with carbon tattooing and a validating ALND, a highly acceptable detection rate and FNR was identified. Residual axillary metastatic burden was low in all false-negative cases. All false-negative cases were operated within the first 2 years of the trial, suggesting a learning curve.

The marking of metastastic axillary lymph nodes and their targeted removal together with SLNB after NST allows higher precision in terms of lower FNRs and higher detection rates. The oncological significance of residual axillary metastases in cases of false-negative SLNB is still unclear, since axillary recurrences after SLNB without completion ALND in individuals converting from cN + to ycN0 are rare [6]. Despite this, the concept of removing the previously affected lymph node with relative certainty is highly attractive. In SLNB alone after NST in initially cN + patients, it is suspected that fibrosis of lymph ducts and within lymph nodes may lead to aberrant lymphatic drainage, resulting in the identification of novel post-NST SLNs instead of original SLNs and thus opening for false-negative SLNB results [19]. The relevance of the double strategy of TAD, i.e. aiming at retrieval of both the SLN and the TLN, is supported by the fact that concordance between SLN and TLN is high but not perfect [4]. In the present analysis, the concordance rate was lower than previously reported which would further support the relevance of performing TAD. Since adjuvant treatment recommendations are increasingly dependent on correct assessment of pathological response, a low FNR does not only contribute to a lower risk of residual axillary metastases but importantly also to the appropriate post-neoadjuvant treatment strategy [20, 21].

Even though targeted procedures have gained much attention and their benefit is suggested in many international guidelines, there is still much uncertainty regarding the method of choice for different clinical settings. Whilst radioactive seeds are implemented in some countries, they may not be used due to radiation legislation in other countries. Metallic clips are presently developed in a multitude of shapes, sizes and reflective characteristics in order to improve detection rates, but still require a second localization procedure, by e.g. guidewire or carbon tattooing before axillary staging surgery, or the use of intraoperative ultrasound. Taken together, all targeted procedures come with an extra cost and the necessity of additional resources, and especially in settings with limited resources, a low-cost strategy with well-documented accuracy would be highly valuable. Therefore, the evaluation of carbon tattooing for the marking of metastastic lymph nodes is clinically relevant and needs to include the assessment not only of feasibility but also of FNR.

The carbon tattooing technique is probably the only marking method in use today that relies entirely on visual identification without any signal given by a probe. Whilst this is an advantage in terms of necessary equipment and cost, it is a potential challenge especially in the voluminous and deep axilla and should be able to cause more damage to lymphatics than probe-based methods due to more extensive surgical dissection. In the present analysis, this problem is probably reflected in the fact that carbon-marked tissue was not always confirmed to contain lymph node structures, suggesting that identification may have been faulty.

The important strength of the present analysis of the TATTOO trial is the use of prospectively collected data, the multicentre setting and the large sample size. In addition, all surgical and histopathological reports were individually scrutinized prior to this analysis in order to identify cases of TLN not containing lymphoid tissue, and cases where the TLN was identified only after the completion ALND. The latter is highly important since retrospective analysis may not yield a realistic picture of the feasibility of TAD itself if it allows detection of the TLN within the ALND sample. Potential limitations comprise the lack of comparison with other marking methods, the inclusion of higher clinical nodal stages that would probably not be eligible for TAD in some guidelines, and the lack of data on previous skin tattoos in the ipsilateral arm in included patients.

## Data Availability

The dataset generated and analysed during the current trial is available from the corresponding author on reasonable request.
